# Perceiving “Complex Autonomous Systems” in Symmetry Dynamics: Elementary Coordination Embedding in Circadian Cycles

**DOI:** 10.3390/ijerph20010166

**Published:** 2022-12-22

**Authors:** Chulwook Park, Jean Hwang, Jae Woong Ahn, Yu Jin Park

**Affiliations:** 1 Institute of Sport Science, Seoul National University, Seoul 08826, Republic of Korea; 2 International Institute for Applied Systems Analysis (IIASA), A-2361 Laxenburg, Austria; 3 Okinawa Institute of Science and Technology (OIST), m1919-1, Okinawa 904-0495, Japan; 4 Department of Physical Education, College of Education, Jeonbuk National University, Jeonju 54896, Republic of Korea

**Keywords:** elementary coordination, circadian rhythm, symmetry breaking, context dependency

## Abstract

This study explored the biological autonomy and control of function in circumstances that assessed the presumed relationship of an organism with an environmental cycle. An understanding of this behavior appeals to the organism–environment system rather than just the organism. Therefore, we sought to uncover the laws underlying end-directed capabilities by measuring biological characteristics (motor synchrony) in an environmental cycle (circadian temperature). We found that the typical elementary coordination (bimanual) stability measure varied significantly as a function of the day–night temperature cycle. While circadian effects under artificially manipulated temperatures were not straightforward during the day–night temperature cycle, the circadian effect divided by the ordinary circadian rhythm remained constant during the day–night cycle. Our observation of this direct, robust relationship between the biological characteristics (body temperature and motor synchrony) and environmental processes (circadian temperature cycle) could mirror the adaptation of our biological system to the environment.

## 1. Introduction

**Figure 1:**
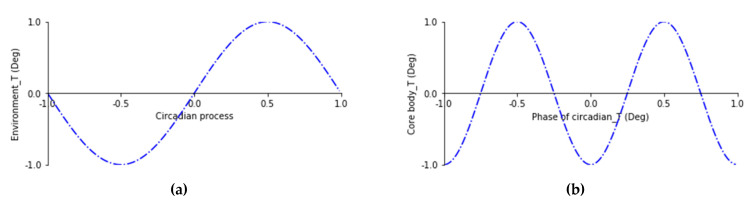
Representation of the circadian rhythm: (**a**) circadian process oscillation, (**b**) temperature (°C) process oscillation between the circadian temperature (horizontal axis) and the body temperature (vertical axis). Note: This is a normalized rhythm, although not all rhythms are identical. Our core body temperature is roughly linked to this cycle, with various hormones released at specific stages in the rhythm because our energy levels are reflected in our body temperature.

The core cycles of the biological system (circadian rhythm) are influenced by 24 h light–dark (environmental) oscillations. Biochemical, physiological, or behavioral processes persist under constant conditions for ~24 h [1]. Presumably due to inputs from the body core to the thermoregulatory centers [2,3], an organism’s circadian rhythm shows a minimum temperature at 05:00 h (when the core body temperature rises most rapidly) but has a more clearly defined maximum temperature at about 17:00 h (when the core body temperature falls most rapidly) in a day–night cycle [4,5] (see Figure 1).

This circadian change (core temperature) is most likely due to rhythmic input from the suprachiasmatic nuclei (SCN) acting on the hypothalamic thermoregulatory centers and altering the thresholds of cutaneous vasodilatation and sweating [6]. Specifically, melatonin appears to contribute to this change; its secretion rate increases in the evening, promoting a decrease in body temperature via cutaneous vasodilatation [7]. Most people are familiar with the amount by which such a process can fluctuate, and how such fluctuations relate to interactions between internal (biological) and external (environmental) circumstances [8]. There is ample evidence of the effects of ecological climate on flora and fauna [9]. Heat exchanged with the environment via convection and radiation influences the gradient between the body core and the temperature [10]. The rhythm in the core temperature produced by this change is normally promoted by other rhythms, including the body clock, sleep, and physical and mental activity, raising the possibility that the disruption of the circadian rhythm could have adverse health consequences [1]. Changes in the body’s interior temperature (not its peripheral or core temperature) are mainly due to circadian rhythmic changes in the rates of ecological impact in animals, including humans [9].

However, we do not yet fully understand the precise control of the internal substance (i.e., SCN) as a generator of the biological circadian rhythm. The circadian rhythm of the core body temperature appears to be generated by periodic variations in heat production and loss [11]. For instance, changes in heat loss via convection and radiation are primarily caused by variations in skin blood flow with consequent changes in skin temperature [5]. In particular, when people are performing mild activities, their decreased temperature is not matched by their thermal load; we can use this observation to describe the thermal responses to activities conducted at different times of the day [12]. Aldemir et al. (2000) found that the submaximal activity changes following the same amount of moderate exercise differed depending on the time of day—that is, the point in the circadian rhythm [13]. The mechanisms responsible for such variations in the core and musculature temperatures during daylight cycles, as a result of normal or non-normal ambient temperatures, alter a range of performance factors, including the thermoregulatory response to activity. These results support the hypothesis that the circadian rhythm influences thermoregulatory responses, and indicate that this hypothesis applies to biological intelligence about certain ecological variables.

### 1.1. Modeling Thermoregulatory Symmetry Breaking

This study investigated the intact movement of a limb oscillator [14] in terms of elementary variations characterized by a pendulum’s dimension [15,16] to harmonize the effects of environmental influence on a well-suited biological model. We consider basic human actions as prime examples of defining complex behavior with simple underlying mechanisms, where different body segments (fingers, arms, legs, head, etc.) are moved, and many of these modes possess some degree of symmetry [17] (Collins and Stewart, 1993).

(1) θ2–θ1 ≈0, θ2–θ1 ≈π

where θ2–θ1 ≈0 denotes a condition of nearly synchronized in-phase, and θ2–θ1 ≈π indicates this in an anti-phase. The property of these dynamics is the oscillation coordination characteristic of our fundamental motor behavior, and constitutes the basis for explaining the regular locomotion of the limbs [18] (Kay et al., 1987). The observed phase relationship (ϕ) between the two oscillators at ϕ≈0° (in-phase) or ϕ≈180° (anti-phase) has been modeled as a point attractor in the limb system, as they are purely stable patterns [19]. This is because routine coordination (e.g., grasping, twisting, and manipulating) involves our limbs, and occurs in various ways owing to the voluntary (or imposed implementation of) numerous interactions [20] (Park, 2022). Moreover, in the observed relative rhythmic segment patterns, the in-phase ϕ=0 condition was more stable than the anti-phase ϕ=π condition [21]. Hence, we applied the elementary synchrony (in-phase) pattern as a reference for a biological system inspired by many studies on 1:1 frequency locking; the difference of the phase (ϕ) in both sides (i.e., θ2 = right limb, θ1 = left limb) right (R) and left (L) helped identify important invariant human movements, muscles, and neural network features [22,23].

(2) $$V\left(\phi \right)=-\alpha \;cos\left(\phi \right)-b\;\cos\left(2\phi \right)$$

The model described in Equation (1) has been adapted in various coupled and uncoupled frequencies in terms of their difference, taking inspiration from the complement of the circadian influences, as given below.

(3) c=circadian temperature cycle, d=core body temperature cycle

where *d* is the preferred rhythmic frequency of homeostasis, and is the circadian cycle of the individual. While $-\alpha \;cos\left(\phi \right)-b\;\cos\left(2\phi \right)$ denotes the strength of the elementary relative phase equilibration (see Section S1 for details), small values of c and d could break the symmetry of the elementary coordination dynamics, excluding their essential coupling characteristics.

(4) $$\left|c\;and\;d\right|>0,\;\left|c\;and\;d\right|\;\approx 0$$

In the proposed assumption, the coefficient of *d* must hold more importance than c to produce the empirically observed perturbation in the equilibrium phase state, and *c* should be set to zero without loss of generality, considering that the environmental circadian cycle cannot be manipulated. If the coupling between *d* and *c* is strong ($\left|c\;and\;d\right|\;\approx 0$), we would expect this pattern to be perfectly symmetrical for environmental requirements. However, in this case, the in-phase rhythmic oscillations in the preferred condition change ($\left|c\;and\;d\right|>0$), and the expected stability or variability of the rhythmical component of oscillation dynamics increases. Therefore, the coordination of the synchrony stability can be extended with a novel symmetry-breaking process, leading to the available effects of bimanual dynamics on circadian temperatures.

(5) $$\dot{\phi }=\Delta \omega -\left[\alpha \;sin\left(\phi \right)+2b\;\sin\left(2\phi \right)]-[c\;sin\left(\phi^{℃}\right)+2d\;\sin\left(2\phi^{℃}\right)\right]+\sqrt{\varrho \xi_{t}}$$

In Equation (2), the symmetric coupling coefficients differ because the bimanual 1:1 rhythmic coordination is performed at different coupled frequencies. This reflects the attractor strengths at 0 and π decreasing instead of the detuning (Δω) increasing. At the same time, the question arises as to what can be expected regarding the apparently similar symmetry temperature case (Δω = 0: core body and circadian cycle parameters). The final estimation between the relative phases of the two oscillators ($\dot{\phi }$) is captured mainly by the parameter of the asymmetric thermoregulatory coupling [$c\;sin(\phi^{℃})+2d\;\sin(2\phi^{℃})]$ with noise ($\sqrt{\varrho \xi_{t}}$).

From this dynamic, we can estimate the different noise types of the underlying subsystems (neural, muscular, and vascular) around an equilibrium point. This suggests that breaking symmetry can be another remarkable feature of the coordinative system. Furthermore, there is no basis for predicting the deviation produced by detuning between thermal manipulation (biological and environmental temperature cycles). Therefore, when devising operational definitions of categories, we must consider the thermal variables of the relative phase frequencies to conceptualize a model.

This request to have the experimental condition of in-phase (ϕ=0) oscillation identical at 1:1 frequency locking (same tempo) can also be fulfilled using the functional symmetry dynamics of different effectors influenced by breaking the asymmetric thermal regulation symmetry through both the circadian temperature cycles. This implies that the effect of one contralateral homologous relative limb phase might not be identical to the others. The expected stability pattern from intuition, given a different motor, suggests an understanding of biological symmetry dynamics in a systemic context of the circadian temperature property.

### 1.2. Purpose and Hypotheses

We aimed to develop an interacting cyclic process for new emergence entities to determine whether something akin to unintentional coordination—an environmental rhythm within an individual’s field of view—would be influenced by a temporal structure irrelevant to the task. We proposed the following hypotheses:

**Hypothesis** **1** **(H1).** *The synchronous coordination between contralateral limbs is captured similarly according to the changes during the circadian temperature cycles*.

**Hypothesis** **2** **(H2).** *The synchronous coordination between contralateral limbs is captured differently according to changes in the thermal (temperature) symmetry breaking between the core body and the circadian state*.

A variety of measures (e.g., phase shift, synchrony, variability, and correlation) will be used to test the influence of environmental conditions and perturbations on the embedding rhythm. We are interested in the main effect of circadian rhythm (α), thermal manipulation (perturbation) (β), and the interaction between the two (α × β) on the dependent variables, namely, the rate of motoric coordination. These relationships will create a specific type of agency guided by what is physically possible. They provide a signpost that addresses the self-potential of the emergence of systems dynamics, suggesting a broader hypothesis for further research on the effects of organism–environment dynamics.

## 2. Experiment I

We designed Experiment I to verify whether ecology influenced the biological scale. To determine the rate of motor synchrony depending on environmental cycles, we conducted an analysis of variance (ANOVA) test comparing normal day–night temperature effects (four circadian rhythm levels: 05:00, 12:00, 17:00, and 00:00) (see Table 1). This addresses the question of whether our system is influenced by an ecological feature. In-phase bimanual coordination synchrony served as a dependent variable, according to the independent variable of normal circadian temperature cycles. This study had eight participants (men = six, women = two, ages 25 ± 3 years).

### 2.1. Experiment I Apparatus and Procedure

We performed the in-phase coordination assessment without detuning with the participants seated in chairs and holding the pendulums vertically, without vision occlusion. Our pendulums were two standard wooden rods (weight 85 g, length 1 m, diameter 1.2 cm), each with a DC potentiometer attached at the top and a 200 g weight attached 30 cm from the bottom. We asked each participant to firmly grasp the pendulums at a point 60 cm from the bottom, asking them not to let the pendulums slip and not to rotate their finger joints. We fixed their forearms voluntarily to restrict the pendulum motion to the sagittal parallel plane and the joint vertical axes (i.e., each oscillation pertained to only one joint, with the other joints immobile). We conducted the experiment sessions by tapping into an ongoing circadian rhythm, focusing on the thermal structure, at four temperature (normal) embedding cycles (05:00, 12:00, 17:00, and 00:00). Each participant completed four sessions with six trials per session (1 participant, 1 min, 24 trials = 6 trials × 4 circadian point sessions). Each trial block lasted 1 min, with a rest period of 5 min. We gave the participants instructions about the preferred pendulum.

### 2.2. Experiment I Proposed Analyses

Research tells us that the in-phase ϕ=0 is more stable than the anti-phase ϕ=π, leading to the identification of important invariant human system features. We analyzed the in-phase bimanual coordination according to the natural period of a pendulum system in all experiments (see Section S2: Calculating Relative Phase Coordination Dynamics).

### 2.3. Experiment I Statistical Data Testing for the Hypotheses

We analyzed a statistical measurement of the *F* distribution for the first potential of circadian embedding in biological cycles, estimating the values of “$\phi_{ave-}\;\phi_{0}$” (fixed-point shift), “$SD\phi \left(rad\right)$” (variability as a function of frequency competition), and “$H\left(x\right)$” (see Section S3: Entropy Production for more details) in the following null and alternative hypotheses.

(6) $$H_{0}:\;\theta_{C}=\theta_{B},\;H_{1}:\;\theta_{C}\neq \theta_{B}$$

Because this is a typical strategy of estimating whether a certain hypothesis is true, we could use statistics to prove the nonexistence ($\theta_{C}=\theta_{B}$) or existence ($\theta_{C}\neq \theta_{B}$) of a relationship. This was a generalization that the circadian component would affect the biological component by dividing the difference between the circadian time point means (variance between) by the difference between the participants within the circadian time point (variance within), as follows:

(7) $$F=\frac{MC_{between}}{MC_{within}}$$

### 2.4. Experiment I Results

**Figure 2:**
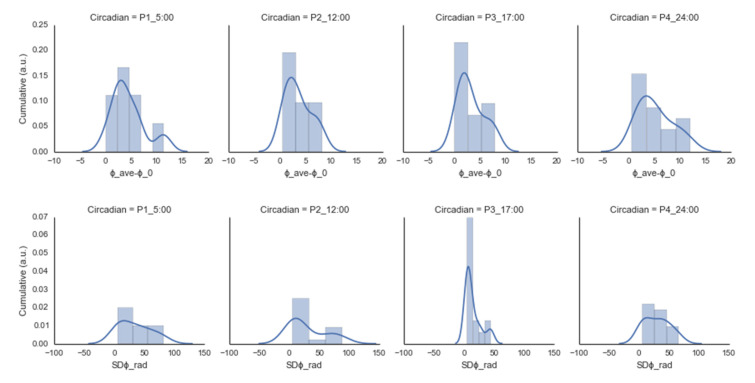
Tendency of the performance parameters in the normal circadian condition (P1–P4 = arbitrary number for assigning an order): $\phi_{ave-}\;\phi_{0}\left(rad\right)$ = fixed-point shift, SDϕ = variability as a function of the frequency competition with an arbitrary unit (a.u.) and density function (cumulative).

This study’s main objective was to provide an explicit demonstration of how parameter dynamics might affect how much information decays. Specifically, we used empirical methods pertaining to how thermodynamic variables (temperature) affect the emergence of order and collective behavior (relative phase) in systems, analyzing the data from the perspective of stability (including the maximal entropy production rate) during the order–disorder transition of a biophysical system. To achieve this, we asked the participants to swing the pendulums with in-phase oscillations at different joint points (three levels) following a metronome beep (in-phase 1:1 frequency locking at 1.21 s), but we only gathered the wrist joint data for the analysis (see Section S4 for details). Each participant completed four sessions with six trials (1 participant, 1 min, 24 trials = 6 trials × 4 circadian temperature sessions) (see Table 2 and Figure 2).

**Figure 3:**
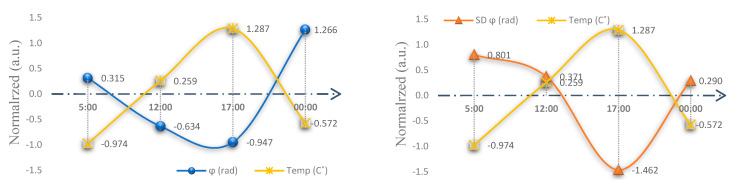
General tendencies in the normal condition: Normalized = standard score (Z calculation); a.u. = arbitrary unit; $\phi_{ave-}\;\phi_{0}$ = fixed-point shift; SDϕ = variability as a function of the frequency competition; Temp = temperature (Celsius); 5 = 5:00 h, 12 = 12:00 h, 17 = 17:00 h, and 00 = 00:00 h. Note: The core body temperature rhythm was at a minimum at 05:00 h and at a maximum at approximately 17:00 h; behavioral performance (variability) was at a maximum at 05:00 h but its minimum was more clearly defined at approximately 17:00 h.

Figure 2 shows the participants’ average performance tendencies in the ordinary circadian cycle, indicating the differences in each parameter. It shows that the main effect of variability [$SD\phi \left(rad\right)$] was not significant (*F*(1, 3) = 1.233, (*p* < 0.316)), nor was the fixed-point shift ($\phi_{ave-}\;\phi_{0}$) (*F*(1, 3) = 1.226, (*p* < 0.319)). However, normalization revealed differences in the circadian cycle widths, especially in the variability of the circadian 5:00 and 17:00 points (t = 2.043, *p* < 0.060) (see Table 3 and Figure 3).

The opposite directions between the temperature and parameter (see note in Figure 3) suggest that our core body temperature cycles are influenced by the surrounding environmental temperature cycles with a 24 h light–dark oscillation, whereas behavioral processes persist under ordinary conditions with a period length of ~24 h.

## 3. Experiment II

For Experiment II, in-phase bimanual coordination synchrony served as a dependent variable to two independent variables: two levels of circadian rhythm and two levels of thermal variable manipulation. Regarding the irregular thermal structure, we examined the temperature-perturbed day–night circadian effects at dawn (05:00) and dusk (17:00), considering that our core temperature reaches its maximum at approximately these times [4] (see Table 4).

### 3.1. Experiment II Apparatus and Procedure

We performed in-phase coordination without detuning with the participants seated in chairs and holding the pendulums vertically without vision occlusion, as in Experiment I. The sessions were introduced for short-term thermal variable manipulation involving two conditions (normal and abnormal), with two temperature embedding cycles (5:00 h and 17:00 h, the lowest/highest peak of the circadian rhythm of the core temperature with skin capacitance). Each trial block lasted 1 min, followed by a 5 min rest. The trial and rest times were related to the maintenance of the thermal capacity of the body [24]. In the natural session (normal temperature), the participants received instructions regarding the preferred pendulum movements, as in Experiment I. For the perturbed condition (abnormal temperature; artificially increasing temperature), the participants wore heat vests that increased their core temperatures for 30 min. We checked their core temperatures using two sensors, a HuBDIC HFS-100 noncontact temperature sensing device and a CORE body temperature monitoring device, neither of which was impacted by environmental thermal influences, and thus provided medical-grade accuracy according to ISO_80601-2-56 (both devices were purchased from CORE Co., Ltd.). After 30 min, we verified the temperature change and collected the data for each trial in the same manner used in the natural condition setup. Because we had concerns about body temperature changes, we checked the participants’ temperature during each rest period. To prevent any additional effects from motion during the trial, we chose intermittent movements for a short time, considering the participants’ body temperature capacities, which were lowered using an ice vest and exogenous temperature (within 30 min) [25]. The entire session for each block lasted a maximum of 30 min (see Section S5 for details).

### 3.2. Experiment II Proposed Analyses

Our analysis, conducted as in Experiment I, raised the question of whether our systems can adapt to a regular or irregular thermal structure. The measurement identified several ways this might be possible. For example, energy can be distributed in a different system, and we can estimate the value of “$\phi_{ave-}\;\phi_{0}$” (fixed-point shift), “$SD\phi \left(rad\right)$” (variation), and “$H\left(x\right)$” (system disorder) using the following null and alternative hypotheses.

(8) $$H_{0}:\;\theta_{A}=\theta_{B},\;H_{a}:\;\theta_{A}\neq \theta_{B}$$

The statistical test of the experimental condition with components of $\theta_{A}$ and $\theta_{B}$ involves monotonic mapping onto the measurement variable $\dot{\phi }$. We established a hypothesis in which different experimental conditions of the external source (circadian temperatures) have a significant effect on the internal source or source of force (bimanual motor variable) of $\dot{\phi }$. Specifically, different external components of the circadian processes or temperature have a significant effect on the degree of internal stability. The internal perturbation from an external source will have a significant effect on the biological entropy of “H”.

(9) $$F_{\alpha }=\frac{MS_{\alpha }}{MS_{between}},\;F_{\beta }=\frac{MS_{\beta }}{MS_{between}},\;F_{\alpha \times \beta }=\frac{MS_{\alpha \times \beta }}{MS_{between}}$$

At this point in the study, we calculated the statistical *F* by dividing the difference between groups ($MS_{between}$) by the difference between subjects within the group under investigation (MS). We observed a main effect of circadian rhythm (α), thermal variable manipulation (temperature perturbation) (β), and the interaction between circadian rhythm and thermal variable manipulation (α×β) on the dependent variable of entropy production. Thus, we performed statistical testing to compare the *F* distribution associated with each item of interest to the error variance to determine whether each effect was meaningful.

### 3.3. Experiment II Results

**Figure 4:**
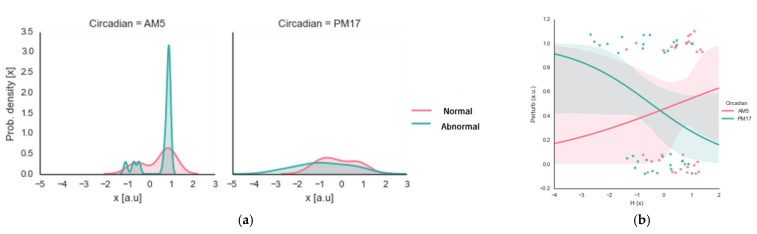
Circadian and temperature perturbation (heat-based)-dependent influences: (**a**) shows the data double-plotted with the variation in a histogram and the kernel density function (vertical axis = probability density, horizontal axis = entropy production; False = normal, True = perturbation; Circadian AM = 5:00 in the morning, Circadian PM = 5:00 in the afternoon); (**b**) shows the relationships between the entropy (horizontal axis) and perturbation (vertical axis) for the different circadian points (red line and dots denote 5:00 h; blue line and dots denote 17:00 h).

We used data collected from the participants (men = six, women = two, ages 25 ± 3 years) for the abnormal (heat-based) day–night circadian temperature effects. However, we collected only the wrist joint data in terms of entropy production. We analyzed each participant’s data four times, with six trials each time. Considering that the body’s core temperature reaches its maximum at approximately 17:00 h and minimum at approximately 05:00 h [4], we compared the day–night circadian temperature effects from the dawn (05:00 h) and dusk (17:00 h) data, including the (ab)normal temperature effects. In the perturbed condition, prior to actual data collection, the participants (*n* = 8) wore heat vests for 30 min to increase their core temperatures. This additional data collection enabled us to compare the perturbed data with the previous normal data (1 participant, 1 min, 24 trials = 6 trials × 2 circadian point sessions (normal dataset) × 2 (temperature perturbation dataset) to account for core body temperature increase due to the heat vest) (see Table 5 and Figure 4).

Figure 4 shows the bimanual coordination stability of the circadian time point, including the temperature perturbation (artificially increased core body temperature due to the heat vest). As shown in Table 5, the main effect of the circadian rhythm was *F*(1, 3) = 20.531 (*p* < 0.001); the temperature perturbation’s main effect was *F*(1, 3) = 1.301 (*p* < 0.258); and the temperature perturbation by the circadian rhythm was *F*(1, 3) = 3.453 (*p* < 0.068). These results indicate that although the participants exhibited significantly greater entropy levels at 05:00 h than at 17:00 h in both conditions (circadian effect), the system disorder measured by the entropy production between the morning and afternoon was exaggerated when we artificially increased the participants’ body temperature (interaction effect).

## 4. Experiment III

For Experiment 3, the sessions were introduced for short-term thermal variable manipulation involving two conditions (normal and abnormal), with two temperature embedding cycles (05:00 h and 17:00 h, the lowest/highest peak of the circadian rhythm of core temperature with skin capacitance). As in Experiment 2, we examined the data from dawn (05:00 h) and dusk (17:00 h) because our core temperature reaches its maximum at approximately 17:00 h and minimum at approximately 05:00 h (see Table 6).

Again, in-phase bimanual coordination synchrony served as a dependent variable to two independent variables: two levels of circadian rhythm × two levels of thermal variable manipulation (normal and decreasing).

### 4.1. Experiment III Apparatus and Procedure

We used the same procedures and apparatus as in Experiment II. However, for the perturbed condition, the participants wore ice vests that decreased their core temperatures for 30 min (see Experiment II Apparatus and Procedure and Section S5 for details).

### 4.2. Experiment III Proposed Analyses

We performed the same statistical testing and analysis as in Experiments I and II (see their respective Proposed Analysis sections).

### 4.3. Experiment III Results

**Figure 5:**
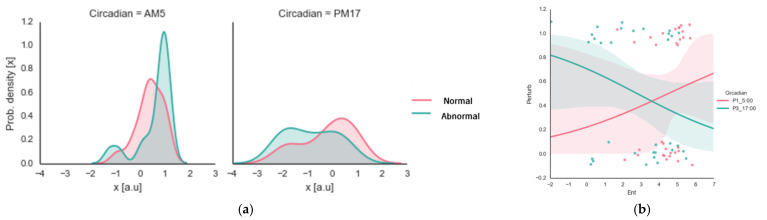
Circadian and temperature perturbation (ice-based) dependent influences: (**a**) shows the data double-plotted with the variation in a histogram and the kernel density function (vertical_axis = probability density, horizontal_axis = entropy production; False = normal, True = perturbation; Circadian AM = 5:00 in the morning, Circadian PM = 5:00 in the afternoon); (**b**) shows the relationships between the entropy (horizontal axis) and perturbation (vertical axis) for the different circadian points (red line and dots denote 05:00 h, blue line and dots denote 17:00 h).

For the abnormal (ice-based) day–night circadian temperature effects, we collected data collected from the participants (men = five, women = three, ages 25 ± 3 years). However, we only calculated the wrist joint data with regard to entropy production. We analyzed each participant’s data four times, with six trials each time. We used data collected at dawn (05:00 h) and dusk (17:00 h) for the circadian temperature effect, considering that our core temperature reaches its maximum at those times [4]. In the perturbed condition, prior to actual data collection, the participants (*n* = 8) donned ice vests for 30 min to increase their temperature by a few degrees. This additional data collection enabled us to compare the perturbed data with the previous normal data (1 participant, 1 min, 24 trials = 6 trials × 2 circadian point sessions (normal dataset) × 2 (temperature perturbation dataset) to account for core body temperature decrease due to the ice vest) (see Table 7 and Figure 5).

Figure 5 shows that the significant circadian main effect was *F*(1, 3) = 23.041 (*p* < 0.001); the temperature perturbations effect was *F*(1, 3) = 1.211 (*p* < 0.275); and the temperature perturbation by the circadian cycle was *F*(1, 3) = 4.264 (*p* < 0.043). These results also indicate (as in Figure 4) that although the participants exhibited significantly greater levels of entropy levels at 05:00 h than at 17:00 h in both conditions (circadian effect), the system stability associated with temperature between the morning and afternoon was exaggerated when the body temperature was artificially decreased (interaction effect).

## 5. Discussion

The present study measured the biological properties of circadian rhythms. After determining the most relevant internal source (pilot test: see Section S4 for details) as a typical dependent variable, we derived the in-phase coordination of the two pendulums (relative phase *ϕ* = 0°) (Experiment I (*n* = 8)) with no detuning (i.e., the two pendulums had the same eigenfrequency). In Experiment I, participants performed bimanual coordination at a fixed-paced metronome rhythm. We examined a variety of measures (e.g., fixed-phase shift, variability, and entropy) for evidence of entrainment or any influence of the embedding rhythm on stability or attractor location. The results show differences between the embedded effects of the light and dark portions of the cycle, revealing that our biological system follows a temperature-embedded day/night environmental system.

Experiments II and III focused on the thermal structure of circadian rhythm. At dawn (05:00 h) and dusk (17:00 h), we performed in-phase coordination without detuning in a short-term thermodynamic manipulation condition. We used a metronome to set an oscillation interval that reflected the natural period of the pendulum system. Consistent with the standard body temperature cycle presented in the results, the core temperatures of the participants were higher at 17:00 h than at 05:00 h. In addition, the ice/heat vests affected the temperature more at 05:00 h than at 17:00 h. We estimated the dynamics of the relative phase between the two limbs (oscillating at the wrists) for system disorder (i.e., entropy production). The influence of the vests was negatively exaggerated (increasing uncertainty) at dawn and positively exaggerated (decreasing uncertainty) in the evening. The results from this biological scale correspond to the theoretical suggestion that the dynamics of the relative phase between the two oscillating limbs were affected by the temporal locus during the circadian cycle; the nonequilibrium phase transition rate of entropy production varied corresponding to the new energy source involved [26]. This indicates that access differed as a function of the circadian cycle, and it can be manipulated using temporary thermal interventions (e.g., ice/heat vests).

Researchers have studied the intelligence from “outside the head” of an organism and the environment, considering the achievements of perceiving and acting as continuous processes [27]. This is an active and autonomous regulation process even in single-cell organisms [28]. Contemporary research has examined exhibitions of autonomy and function control to explain agencies scientifically [29,30]. The key reasons for the existence of multiple behavioral modes and the simplicity of switching among the modes have been established [31]. However, resource availability makes investigation challenging. Therefore, our results from empirical evidence might answer the following question: how can we define behavior in terms of how organisms function? We sought to understand this directed behavior as a result of the workings of the organism–environment system rather than as simply pertaining to the organism [32]. Therefore, we sought to expose the laws that underlie homeostatic regulation; these might be both cyclic and adaptive, supporting the finding that access differs as a function of the circadian cycle and can be manipulated by temporary thermal interventions.

### 5.1. Practical Implications

Some research has suggested that people generally perform better at tasks such as mental arithmetic in the early morning [33,34]. However, another study found an evening peak for this type of performance in highly practiced young adults [35]. With a low working memory load, performance is positively correlated with the circadian rhythm of body temperature [36]. The majority of performance-related components (e.g., flexibility, muscle strength, and short-term memory) vary depending on the time of day. Contemporary models of subjective alertness and performance efficiency view these variables as being determined both by a homeostatic process (number of hours since waking) and by inputs from the circadian timing system [37]. Much more research is required for us to understand which performance tasks exhibit different time-of-day effects.

In line with previous evidence related to performance quality dependent on organism–environment interactions, the present study determined the impact of circadian misalignment on biological functions. It also raised the possibility that disrupting circadian systems might induce physical complications. The self-attunement mechanism of current performance affects many nested and interconnected scales, not just a single component. These interdependencies in different physical object phases suggest a context-dependent explanation for goal-oriented movements and the emergent assumption of the principle of organisms embedded in their environmental contexts while considering the infinite distinct representations of the system’s productivities.

### 5.2. Limitations and Suggestions for Future Study

The present study had several limitations that should be addressed in future studies to strengthen the findings’ applicability. First, our study used data collected during one season, which limits its generalizability; the findings might not apply to all seasons. For instance, the moderating effect of biological variability might be more (or less) marked in indoor (or outdoor) environments that closely resemble (differ from) the body’s normal temperatures. Thus, the present study should be replicated across different conditions (different seasons, indoor/outdoor, different altitudes, etc.) to ensure generalizability.

Second, although we considered the experimental group as an identical population in terms of general demographic characteristics (i.e., all were university students of similar ages), the independence and individualistic tasks were independent of the moderating effect of temperature cycle variability. Hence, these factors could still be substantially mediated by individual lifestyle tendencies in creating a biological measurement scale. Moreover, the study did not consider other potential individual effects; future studies should consider key boundary conditions for controlling individual effects of biological stability on the relationships between external levels and internal behaviors. For instance, working hours and sleeping habits (i.e., how long participants worked and slept before participating in the experiment) could have interfered with the relationship between the temperature level and biological performance by themselves or in conjunction with the environmental cycle. The uniqueness of the moderating effect of individual tendency variability could be confirmed by examining the effects of the potentially confounding variables.

Third, we controlled the clarity of the temperature perturbation scale items in Experiments II and III. True biological stability is distinct from the device noise caused by vague experimental conditions. If the devices used to present perturbations (the heat and ice vests) can be understood in qualitatively different ways because of their unclear operations, the biological stability of these items can be over- or underestimated, skewing the results. Thus, caution is required in the interpretation and generalization of the results related to Hypothesis 2.

Finally, objective outcome variables related to stability, such as neural/chemical functions and microstate severity, could be used as dependent variables to examine the variant and invariant effects of the temperature cycle instead of behavioral performance-based measurements from participants. We would better understand the practical importance of systematically managing biological adaptations to environmental cycles if we obtained similar findings with these objective neurochemical outcome variables. Future studies should examine the moderating effect of neurological significance on the environmental temperature context level.

## 6. Conclusions

Inquiry into the possibility of relating perception and action to dynamics began in the 1970s with the problem of coordination: could a principled dynamical account be given of fundamental rhythmic capabilities involving multiple joints, muscle scores, and millions of cells? Efforts to address this question invoked the concepts and tools of nonlinear dynamics (e.g., [16]). One useful approach is homeokinetics, the study of complex self-organizing systems [38]. Homeokinetics looks for cycles at all time scales to show how interacting cyclic processes account for the emergence of new entities, many of which are similarly cyclic. The central idea is that cycles interact to create self-replicating living systems that abide by particular cyclicities [39]. Following these cycles is integral to life. Circadian rhythms are cycles of particular prominence in contemporary research on living organisms. Rather than crediting rhythms to “clock genes”, the dynamic approach considers them an emergent system property.

Circadian rhythms are found in most living organisms, including humans, although they must not be confused with biological systems. However, these two factors control biological rhythms interacting in coordination. This has been an influential research topic because humans are a collection of physical, emotional, and performance systems [37]. Contemporary models of subjective alertness and performance efficiency view the variations between systems as being determined both by the homeostatic process (number of hours since waking) and input from the circadian timing system [40]. However, we need more research to understand which performance tasks show different time-of-day effects with various environmental variables and the mechanisms underlying those differences. Corresponding to a theoretical study on the rate of stability of a system and how it can vary when a new energy source is accessed via a nonequilibrium phase transition process [26], our study’s results reconfirm that access differs as a function of circadian temperature [41,42]. This might reflect that the mechanism of the system’s state is not a specific component of special properties, but a general co-activity encompassing all components [31,43].

## Figures and Tables

**Table 1:** Data collection for Experiment 1: eight participants, four circadian points, six trials at each circadian point.

Condition	Participants (*N*)	Circadian Points	Trials at Each Circadian	Task/Rest (min)
Normal	8	5:00 12:00 17:00 00:00	6	1/5

Note: We asked the participants to swing their limbs in-phase at different anatomy points (192 datasets (3 levels: wrist, elbow, and shoulder)), but we only used the wrist joint data (64 sets) in our analyses. The duration of each trial was 1 min, with 5 min rest intervals between trials.

**Table 2:** Raw values for Experiment 1.

Participants (Index)	Circadian 5:00		Circadian 12:00		Circadian 17:00		Circadian 00:00	
	$\phi_{ave-}\;\phi_{0}$	SDϕ	$\phi_{ave-}\;\phi_{0}$	SDϕ	$\phi_{ave-}\;\phi_{0}$	SDϕ	$\phi_{ave-}\;\phi_{0}$	SDϕ
*P1_*I_W_	1.16	0.604	0.89	0.529	1.58	0.676	6.02	4.314
*P2_*I_W_	4.08	4.318	2.88	1.080	5.01	0.625	3.82	1.604
*P3_*I_W_	1.22	0.67	4.04	0.604	1.55	0.540	3.91	0.770
*P4_*I_W_	2.67	7.739	1.76	8.378	6.67	1.01	7.67	0.679
*P5_*I_W_	6.18	4.079	3.06	1.367	2.72	1.134	4.68	2.061
*P6_*I_W_	1.95	3.801	5.68	5.971	5.81	3.331	4.625	4.114
*P7_*I_W_	3.52	4.013	4.47	2.096	0.84	0.617	3.87	5.525
*P8_*I_W_	4.25	1.738	5.57	4.004	1.96	3.598	7.16	4.413

Note: Participants are denoted by the arbitrary labeling 1–8; W denotes the wrist data actually used from three different joint datasets. $\phi_{ave-}\;\phi_{0}\left(rad\right)$ = fixed-point shift, $SD\phi \left(rad\right)$ = variability as a function of the frequency competition. We derived the value of I from the execution of each trial *(*$w_{1}$, $w_{2}$*)*, with the values of these two trials divided by 2.

**Table 3:** Averaged variables from the normal day–night temperature values.

	Circadian 5:00		Circadian 12:00		Circadian 17:00		Circadian 00:00	
	$\phi_{ave-}\;\phi_{0}$	SDϕ	$\phi_{ave-}\;\phi_{0}$	SDϕ	$\phi_{ave-}\;\phi_{0}$	SDϕ	$\phi_{ave-}\;\phi_{0}$	SDϕ
N(I)	8	8	8	8	8	8	8	8
AVER	4.382	3.370	3.546	3.003	3.269	1.441	5.221	2.935
STDEV	3.165	2.351	1.717	2.872	2.229	1.267	1.536	1.870
SES	1.119	0.831	0.607	1.015	0.788	0.448	0.543	0.661
Temp (°C)	36.607		36.834		37.023		36.681	

Note: *N*(*I*) = number of cases indexed by the calculation of *(*$w_{1}$ *+* $w_{2}$/2); AVER = averaged fixed-point shift; STDEV = averaged variability from the standard deviation; SES = standard error score; and Temp = core body temperature (Celsius). Because we collected these data at Seoul National University in Seoul, Korea, the temperature is shown in degrees Celsius.

**Table 4:** Data collection for Experiment 2: two conditions, eight participants, two circadian points, and six trials at each circadian point.

Condition	Participants (*N*)	Circadian Points	Trials at Each Circadian Point	Task/Rest (min)
Normal	8	5:00 17:00	6	1/5
Abnormal (heat-based)	8	5:00 17:00	6	1/5

Note: We asked the participants to swing their limbs in-phase at different anatomy points (192 dataset (3 levels: wrist, elbow, and shoulder)), but we only used the wrist joint data (64 sets) in our analyses. The duration of each trial was 1 min, with 5 min rest intervals between trials.

**Table 5:** Normalized entropy production from normal and abnormal (heat-based) day–night temperature effects.

	Circadian 5:00	Circadian 12:00	Circadian 17:00	Circadian 00:00
N(I)	8	8	8	8
Norm (H)	0.410	−0.165	0.564	−0.809
Vari (H)	0.651	0.664	0.627	0.745
SES	0.230	0.235	0.222	0.264

Note: N(I) = number of cases indexed by the calculation of *(*$w_{1}$ *+* $w_{2}$*/*2); Norm = normalized entropy production; Vari = averaged variability from the entropy production; SES = standard error score (see Section S6 for details on the data).

**Table 6:** Data collection for Experiment 3: two conditions, eight participants, two circadian points, and six trials at each circadian point.

Condition	Participants (*N*)	Circadian Points	Trials at Each Circadian Point	Task/Rest (min)
Normal	8	5:00 17:00	6	1/5
Abnormal (ice-based)	8	5:00 17:00	6	1/5

Note: We asked the participants to swing their limbs in-phase at different anatomy points (192 dataset (3 levels: wrist, elbow, and shoulder)), but we only used the wrist joint data (64 set) for our analyses. The duration of each trial was 1 min, with 5 min rest intervals between trials.

**Table 7:** Normalized entropy production from normal and abnormal (ice-based) day–night temperature effects.

	Circadian 5:00	Circadian 12:00	Circadian 17:00	Circadian 00:00
N(I)	8	8	8	8
Norm (H)	0.404	−0.172	0.608	−0.840
Vari (H)	0.446	1.031	0.518	0.993
SES	0.158	0.365	0.183	0.351

Note: N(I) = number of cases indexed by the calculation of ($w_{1}$ *+* $w_{2}$*/*2); Norm = normalized Entropy production; Vari = averaged variability from the entropy production; SES = standard error score (see Section S5 for details on the data).

## Data Availability

All data and materials are our own. The materials and data used to support findings of this study are included in the Supplementary Materials File.

## Supplementary Materials

The following are available online at https://www.mdpi.com/article/10.3390/ijerph20010166/s1. Section S1: Bimanual Coordination and Symmetry-breaking Modeling Dynamics; Section S2: Calculating Relative Phase Coordination Dynamics; Section S3: Calculating Entropy Production; Section S4: Finding a Relevant In-phase Bimanual Synchrony Variable, Section S5: Normal and Abnormal Day–Night Circadian Temperature Perturbations, Section S6: Collected Dataset for Entropy Production.
