# Supplementary material for: Perceiving “Complex Autonomous Systems” in Symmetry Dynamics: Elementary Coordination Embedding in Circadian Cycles
Source: Int J Environ Res Public Health. 2022 Dec 22;20(1):166. doi: 10.3390/ijerph20010166 (PMC9819880; doi:10.3390/ijerph20010166)
Supplement: Supplementary file 1 [file ijerph-20-00166-s001.zip › ijerph-2007161-supplementary.pdf]

International Journal of
*Environmental Research and Public Health*

**Section S1**

***Bimanual coordination dynamics:*** Formation and retention of propriospecific information indicate the states of muscular-articular links, in which the patterns are constrained by the dynamical criteria of stability pattern. To be specific, let us consider a qualitative physical system such as stiffness, damping, and position over time in a dynamical mass spring system as given below.

$$f(t) = mx'' + bx' + kx \quad (1.1)$$

Here, m is mass, b is friction, and k denotes stiffness. The variable t is time, $\chi$ denotes the position, $\chi'$ is the velocity, and $\chi''$ represents the acceleration. In physics, because damping is generated by a process that dissipates the energy stored in oscillations, the interplay between input and damping can be said to approach a stationary fixed point in the long-time limit.

$$mx'' + bx' + kx = 0 \quad (1.2)$$

Such systems possess a static equilibrium point, which is called a point attractor [Kugler, Kelso, & Turvey, 1980]. The property of this dynamic has been applied not only to a physical system but also to descriptions of the human neuromuscular level [Kay et al., 1987]. This function involves an investigation of the intact movement of a limb oscillator in terms of muscle–joint kinematic variations (kinematic position, velocity, acceleration) over time. The action of swinging two limbs comfortably can be characterised by a pendulum's dimension [Kugler & Turvey, 2015; Turvey, 1990], simplifying the point attractor while restricting it to certain domains of phase space.

$$\theta 2 - \theta 1 \approx 0 \quad (2.1)$$

$$\theta 2 - \theta 1 \approx \pi \quad (2.2)$$

In this equation, with the phase difference, $\theta 2 - \theta 1 \approx 0$ denotes a condition of nearly synchronised in-phase, and $\theta 2 - \theta 1 \approx \pi$ indicates this in an anti-phase. The observed relative phase or phase relation ($\phi$) between two oscillators at $\phi \approx 0°$ (in-phase), or $\phi \approx 180°$ (anti-phase) have been modelled as the point attractors in our limb system, as they are purely stable patterns [Pikovsky et al., 2003].

In the observed relative rhythmic segments patterns, the in-phase $\phi = 0$ condition is more stable than the anti-phase $\phi = \pi$ condition [Kelso et al., 1984]. Inspired by a number of studies on the 1:1 frequency locking of the left-and-right-hand phase defined as $\phi = (\theta_L - \theta_R)$ — the difference between the left (L) and right (R) phase angles ($\phi$) — has led to the identification of important invariant human system features [Zanone & Kelso, 1994; Turvey & Schmidt, 1994].

$$V(\phi) = -\alpha\, cos(\phi) - b\cos(2\phi) \quad (3)$$

In this equation, $\phi$ is the phase angle of the individual oscillator. In addition, $\alpha$ and b are coefficients which denote the strength of the coupling between the two oscillators. The relevant regions of the parameter space allow the potential $V(\phi)$, the negative signs in front of the coefficients simplify the equation of motion. A relative 1:1 frequency-locked coordination phase [$V(\phi)$] can be determined by the differences between the continuous phase angles [$-\alpha\, cos(\phi) - b\cos(2\phi)$] of two component oscillators, and the stability of the point attractor can be varied by varying the pendulum's dimensions [Kugler & Turvey, 2015].

This function indicates that the minima of the potential are located at $\phi$=0, and $\phi = \pm\pi$ [Haken, Kelso, & Bunz, 1985]. Given this scenario, the function can be estimated in how the potential will change in

shape as the control parameter (energy cost) increases. Based on the observed mechanism for the point attractor with a simple function, the present study proposes the in-phase bimanual rhythmic coordination synchrony pattern as a particularly well-suited physical model (see Section S5 for detailed information on the pilot test). This allows a useful reference of system stability coordination tasks in which this functional pattern can be applied to all human movement, muscles, and even a neural network. An actual intersegmental coordination, however, is additionally shaped by the contingencies of adjusting to environmental vagaries. How these extrospecific requirements and information types are incorporated into the physical stability patterns can be determined by the level of symmetry coordination [Amazeen, Amazeen, & Turvey, 1998]. To harmonise the effects of motor stability toward environmental symmetry, this study investigates the following elaboration.

***Symmetry breaking in bimanual coordination dynamics:*** The potential [V(ϕ)] extends the described assumption in terms of the difference between the uncoupled frequencies of bimanual rhythmic components;

$$\Delta\omega = (\omega_L - \omega_R) \quad (4)$$

where $\omega$ is the preferred movement frequency of the left ($\omega_L$) and right ($\omega_R$) individuals. If the relative phase between $\omega_L$ and $\omega_R$ was equal ($\Delta\omega$ = 0), this pattern would be assumed as a perfectly identical symmetry. However, since the preferred movement frequencies of the individual oscillators in the in-phase are large (i.e., function: b/a=0.5, detuning=-0.5, or detuning=-1.5), the expected stability of the rhythmical limb oscillation dynamics becomes greater than equal (see Figure 2).

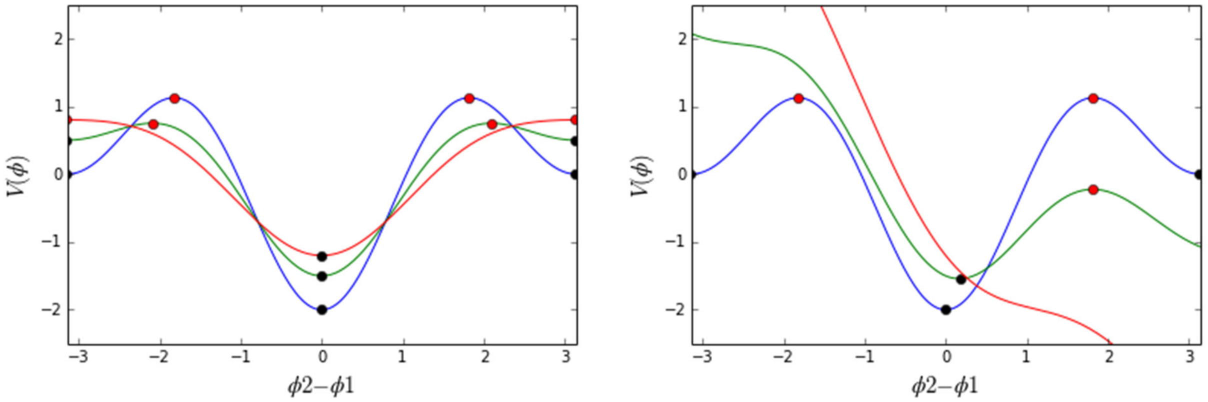


**Figure S1.** Reflection of a potential function (HKB model). Plot on the left: Blue line = the vertical axis denotes the energy of the function at each averaged relative phase. The horizontal axis indicates the averaged relative phase between two limbs from in-phase 0 to anti-phase 3.14 = 180 (-3.14 = -180). At the local point of 0 and 180 (-180), the function is close to the minima (attractors; black balls), and at the local point of approximately 90 (-90), the state is close to the maxima (repellors: red balls). The red and green lines denote the variation of the potential functions. Depending on the preferred frequencies of the two components, the intrinsic dynamics of the potential function of V is determined. Black balls symbolise stable states (attractors) and red balls denote unstable states (repellors). While all states of the systems become less stable due to a higher energy level, the balls at the relative phase point of 0 (in-phase) remain; these balls in the anti-phase condition disappear because the system is shallower. The plot on the right shows the preferred movement frequencies of the individual oscillators. The blue line denotes identical symmetry. The green line denotes large difference in symmetry (function: b/a=0.5, detuning=-0.5). The red line denotes an even larger difference in symmetry (function: b/a=0.5, detuning=-1.5).

Such phenomena of symmetry breaking must be another fundamental feature of the coordinative system [Amazeen, Amazeen, & Turvey, 1998]. From this dynamic, a different noise of the underlying subsystems (neural, muscular, and vascular) can be estimated around an equilibrium point, which may help in conceptualising the model when it comes to making operational definitions of each category that the model has to consider in order to account for the variability of the relative phase frequencies between two limbs;

$$\dot{\phi} = \Delta\omega - \alpha\, cos(\phi) - \text{b}\cos(2\phi) + \sqrt{\varrho\xi_t} \quad (5)$$

The estimation between two oscillators' relative phase ($\dot{\phi}$) is captured by the parameter ($\Delta\omega$) of preferred movement frequency of the individual segment [$\alpha\, cos(\phi) - \text{b}\cos(2\phi)$] with the noise ($\sqrt{\varrho\xi_t}$). Given the equation of the preceding model (grouped as the kinematics of motor stability according to the coordination task of synchronisation), such a term has been used to capture purely functional dynamics regarding the equilibria and confirmed usually as in the time and temporal difference between two oscillating limbs [Treffner & Turvey, 1996].

A study that experimented on handedness [Treffner & Turvey, 1996] advanced the elementary coordination dynamics of Eq. 5. The researchers added two add (sine) terms of the coefficients whose signs and magnitudes determined the degree and direction of asymmetry, as follows.

$$\dot{\phi} = \Delta\omega - [\alpha\, sin(\phi) + 2b\sin(2\phi)] - [c\, sin(\phi) + 2d\sin(2\phi)] + \sqrt{\varrho\xi_t} \quad (6)$$

Here, $\dot{\phi}$ indicates a coordination change. $\Delta\omega$ refers to the breaking of symmetry due to the frequency competition between two limbs. [$\alpha\, sin(\phi) + 2b\sin(2\phi)$] denotes a symmetric coupling defined by a relative phase of 0 and π attractors (this form of the term could be derived as the negative gradient potential V with respect to ϕ), and the [$c\, sin(\phi) + 2d\sin(2\phi)$] term means added asymmetric coupling attractors with the stochastic noise $\sqrt{\varrho\xi_t}$. This extended equation implies that the emergent elementary dynamics between limbs or limb segments was governed by a slightly asymmetric potential of [$c\, sin(\phi) + 2d\sin(2\phi)$], which indicates the extended collective dynamics of inter segmental rhythmic coordination of the periodic components.

**Section S2**

***Calculating relative phase coordination dynamics****:* The mechanism of oscillation on two different (left pendulum and right pendulum) but nearly identical process phases was defined by the following dynamic:

$$\phi = \theta_L - \theta_R \quad (7)$$

Here, $\phi$ is the phase of the strength between the left hand ($\theta_L$) and the right hand ($\theta_R$). The degree of relative phases (0°–180°) depends on the difference in the two oscillators. If each $\theta$ was defined as a sine function, as follows,

$$\theta_1 = \omega_1 - \frac{\alpha}{2}\sin(\theta_1 - \theta_2) \quad (8.1)$$

$$\theta_2 = \omega_2 - \frac{\alpha}{2}\sin(\theta_2 - \theta_1) \quad (8.2)$$

the logic can simply be rewritten to $\phi$ as the same dynamic function.

$$\theta_1 - \theta_2 = \phi \quad (8.3)$$

Then, each $\theta$ can be specified as the following equation:

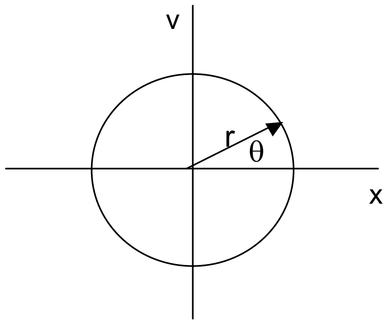


$$\left.\begin{matrix} \dot{r} = 0 \\ \dot{\theta} = \omega \end{matrix}\right\} \rightarrow \theta(t) = \omega t + \theta(0)$$

$$\theta_1 = \omega_1 = \theta_1(t) = \omega_1(t) + \theta_1(0)$$

$$\theta_2 = \omega_2 = \theta_2(t) = \omega_2(t) + \theta_2(0)$$

$$\phi = \theta_2 - \theta_1 = \omega_2 - \omega_1 = \Delta\omega(t) + \phi(0) = \phi(t) \quad (9)$$

In order to realize the relative phase-time series of both hands as calculated with the above logic, it was necessary to find the maximum extensions from the 3D data collected from the DC potentiometers used here. Although the data were fairly regular as the participants were instructed to establish in-phase 1:1 frequency locking at a 1.21 s metronome beat, these were calculated via a principal component analysis to collapse the 3D data values into 1D data values.

First, data from both hands were dividing into left and right components. Next, the separated data were collapsed as each peak point (left_peaks and right_peaks) in terms of a one-dimensional dataset, and these peak points were defined with each vector value to link each peak point according to the time series. Finally, these sets were analysed using a discrete relative phase formula, as follows:

$$\phi_i = 2\pi \frac{t_{max}L_i - t_{max}R_i}{t_{max}L_{i+1} - t_{max}L_i} \quad (10)$$

In this equation, $t_{max}L_i$ is the time of the '$i$'th maximum extension of limb L, and $\phi_i$ is the relative phase for cycle $i$. The phase of relativity time series for both oscillators were established using a direct function of the data analysis program.

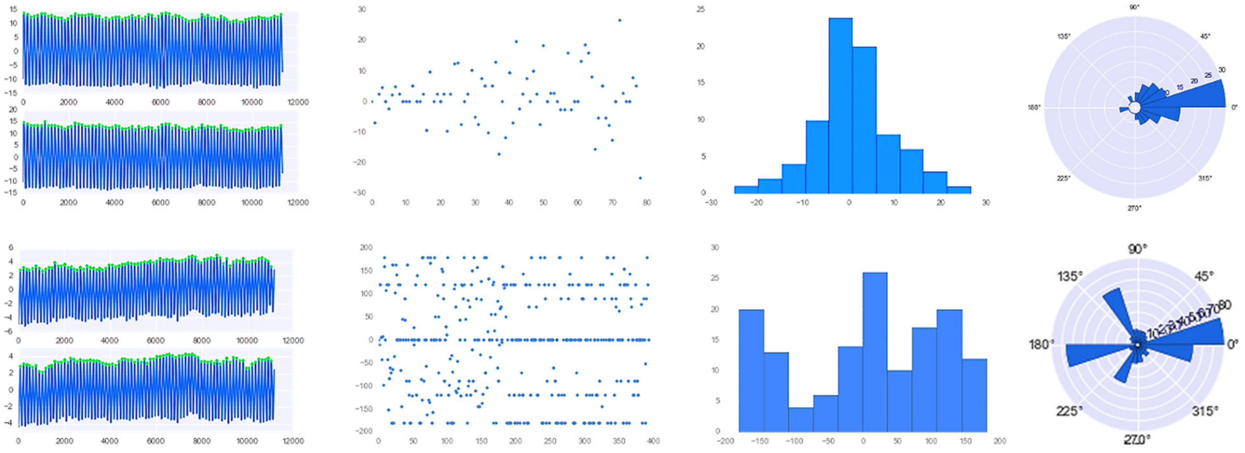

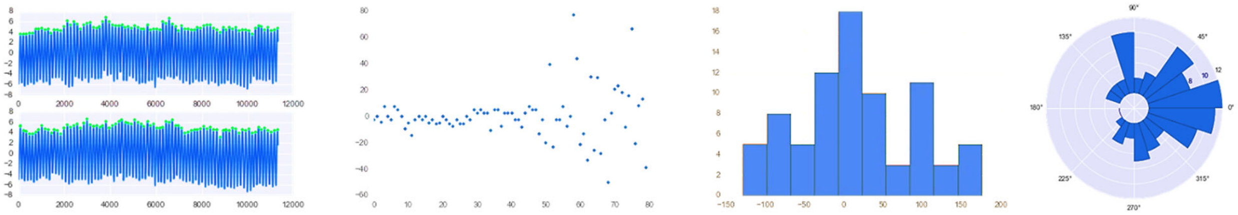

**Figure S2.** Graphical illustration of the inclusion and exclusion criteria (Upper = inclusion case, middle and bottom = exclusion case). To show the exclusion criteria, we added the data on exclusion cases based on the above procedure. As shown in the figure, even if same in-phase 1:1 frequency locking was applied, data from the task were likely contaminated by distractions from participants (bottom Figures) or devices (middle Figures). In such a case based on this basic calculation, we excluded the data from five participants (design 1 = 2, design 2 and 3 = 2, design 4 = 1). The figure on the left denotes the frequency range of the amplitude (horizontal axis = time series and vertical axis = displacement, with the upper figure denoting the left-hand side and the bottom denoting the right-had side). The figure on the middle left illustrates the discrete relative phase synchrony (horizontal axis = time series, vertical axis = relative phase checked peaks). The figure on the right in the middle is a phase histogram [horizontal axis = relative phase (0 equal in phase, ± 180 equal ± anti phase), vertical axis = proportion of the occurrence]. The shaded section in the figure on the right represents the pendulum angle degree and variance [regarding this circular function, we used 2π as the default value (0) and calculated x via (180 degree*x/pi). In the sample case above, the degree represents the degree of closeness to the in-phase (0 degree, or 360 degree) or anti-phase (±180 degree) conditions and the distribution of the joints' relative phases].

Based on the abovementioned procedures, the standard location and relative stability of the stable phase relationships, or fixed points were effectively measured. In these calculations, the locations of the fixed points were indexed as the shift of the mean relative phase from an intended phase.

$$\phi_{ave} - \phi_0(rad) \tag{11}$$

Here, $\phi_0$ is the intended phase of the point shift as a function of the degree of frequency competition ($\Delta\omega = 0$). Although the experimental setting was designed with a 1:1 frequency and in-phase locking with a metronome beat, the mean degree ($\phi_{ave-}\phi_0$) was considered in which the importance of the degree of closeness between the in-phase (0, 360 degree) or anti-phase (± 180 degree) condition with regard to detecting the different conditional phase relationships was represented. For the deviation value, it was necessary to check the datasets for the degree (converted from radian values) of in-phase variability with the following equation:

$$SD\phi(rad) \tag{12}$$

The locations of the fixed points are indexed as the deviation or shift of the mean relative phase $\phi_{ave}$ from the intended phase $\phi$ of the 0. The stability here is inversely related to the variability with which $\phi_{ave}$ is produced, as indexed by the standard deviation of relative phase $SD\phi$.

As noted in equation 5, $\phi$ is the relative phase, $\Delta\omega$ is a detuning parameter, *b*/*a* is a frequency parameter, and the last term denotes noise (fluctuating force). When $\Delta\omega = 0$, the two-oscillator system is relatively symmetrical, but when the symmetry is broken, i.e., when $\Delta\omega \neq 0$, related states appear, which can be described as the difference in preferred frequencies of the two limbs.

$$[\Delta\omega\ (rad * x^{-1})] \tag{13}$$

Here, the left oscillator is $x$ larger than the right one, or vice versa, i.e., $x$ is the size difference that causes the difference in the relative phases between the two oscillators ($\Delta\omega$). Inspired by the traditional asymmetric coupling manipulation between the two oscillators, the main experimental variables of the core body temperature phase and the circadian temperature phase in this study were applied as the functional asymmetry expressed through the detuning term $[c\ sin(\phi^{℃}) + 2d\ \sin(2\phi^{℃})$. Such application assumed that homologous but contralateral phase segments of both limbs will not be identical. Logically, the relativity can be denoted through multiplication by -1, and their solutions are linked to their relative values [-1(a) = (b)]. These simulations with the observations reflect that the fixed point shift can be calculated using the deviation of the average relative phase from the intended phase ($\phi_{ave} - \phi_0$), and the variability can be calculated as the standard deviation of the relative phase ($SD\phi$). This provides us with a basic tool for measuring the system's ability to remain stable in certain circumstances [Amazeen et al., 1997].

## Section S3

***Calculating entropy production***: Application of the average uncertainty model (Shannon, 1984) to actual data set of the experiment.

Let us apply the above calculation to this experimental model. If we have a fair object of $\phi$ of which the symbols have probabilities of 0 degrees = 0.5 and non-0 degrees = 0.5, we would calculate H of x as 0.5 multiplied by the log base 2 of 1 over 0.5, plus 0.5 multiplied by the log base 2 of 1 over 0.5, as follows:

$$\mathrm{H}(\chi) = 0.5\ \times log_2\left(\frac{1}{0.5}\right) +\ 0.5\ \times log_2\left(\frac{1}{0.5}\right) \quad (14)$$

The result is 1. Because

$$\frac{1}{0.5} = 2 \quad (15)$$

and

$$log_2(2) = 1 \quad (16)$$

then

$$\mathrm{H}(\chi) = 0.5 \times 1 + 0.5 \times 1$$
$$\therefore \mathrm{H}(\chi) = 1 \quad (17)$$

In this procedure, the fair object of $\phi$ has one bit of entropy, implicating that when we calculate an object, we will receive an average of one bit of information. However, if the object is not fair, i.e., when the symbols lead us to have probabilities of 0 degrees = 0.75 percent of the time but non-0 degrees = 0.25 percent of the time, the object for our information source will be

$$\mathrm{H}(\chi) = 0.75 \times log_2\left(\frac{1}{0.75}\right) + 0.5 \times log_2\left(\frac{1}{0.25}\right) \quad (18)$$

and the result is approximately 0.811. Because

$$\frac{1}{0.75} = 1.333\ldots, \qquad \frac{1}{0.25} = 4 \quad (19)$$

and

$$log_2(1.333\ldots) = 0.415, \qquad log_2(4) = 2 \quad (20)$$

then

$$\mathrm{H}(\chi) = 0.75 \times 0.415 + 0.25 \times 2$$
$$\therefore \mathrm{H}(\chi) = 0.811 \quad (21)$$

Thus, every time we measure an object of $\phi$ which is not fair, the calculation would give us an average of 0.811 bits of information, indicating that we will receive less (or more) of the information source (or entropy) from this un-faired object of $\phi$ than we would receive from a fair object of $\phi$.

Considering that the actual data set will not be simply two states, if we extend the procedure, one can imagine an object's possibilities, as follows:

$$\{0.1, 0.1, 0.1, 0.5, 0.1, 0.1\}, \text{ or } \{0.16, 0.16, 0.16, 0.16, 0.16, 0.16\}$$

The same number of trials but with different sets of both objects will be approximately as follows:

Left set: $2.1 = 0.1 \times log_2\left(\frac{1}{0.1}\right) + 0.1 \times log_2\left(\frac{1}{0.1}\right) + 0.1 \times log_2\left(\frac{1}{0.1}\right) + 0.5 \times log_2\left(\frac{1}{0.5}\right) + 0.1 \times log_2\left(\frac{1}{0.1}\right) + 0.1 \times log_2\left(\frac{1}{0.1}\right)$

Right set: $2.5 = 0.16 \times log_2\left(\frac{1}{0.16}\right) + 0.16 \times log_2\left(\frac{1}{0.16}\right) + 0.16 \times log_2\left(\frac{1}{0.16}\right) + 0.16 \times log_2\left(\frac{1}{0.16}\right) + 0.16 \times log_2\left(\frac{1}{0.16}\right) + 0.16 \times log_2\left(\frac{1}{0.16}\right)$

This represents on average that we will receive less entropy (Figure on the left = 2.1) from a balanced object than from an unbalanced object (Figure on the right = 2.5).

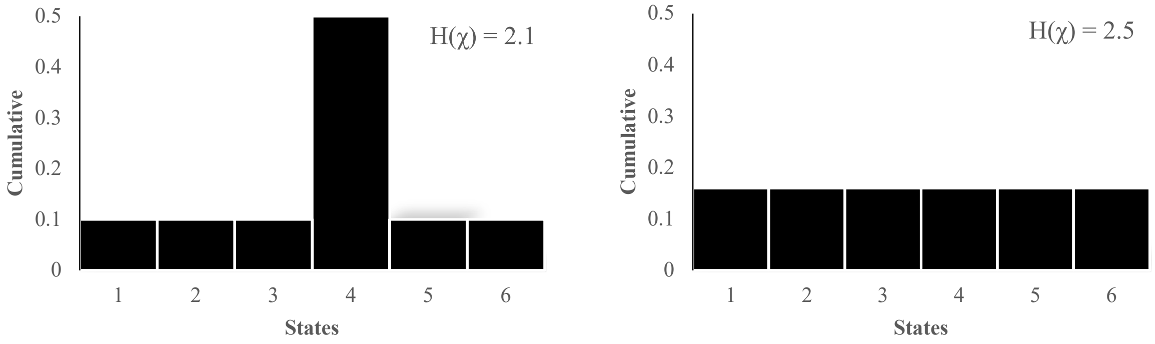


**Figure S3.** Plots show simulation with different objects. The figure on the left denotes an un-faired set while the figure on the right denotes a faired set. Note: Regarding comparison of this simulation with variability, the left set's variability calculated by the way of standard deviation was 0.163, while the right set's variability calculated by the way of standard deviation was 0.000. Such comparison reflects that the uncertainty calculation by the Shannon equation shows us different information in spite of using the same source.

Inspired by this simulation, we defined the probabilities as nonzero relative phase heights (cumulative function) based on an earlier previous elementary coordination calculation (see the previous section entitled "*Calculating relative phase coordination dynamics*") of the artificially given states (20 bins) of the histogram.

This procedure allows us to calculate the uncertainty of an information source with any number of sets, noting that such a formulation used with the above procedures was based on observations made and experiments done on a macroscopic dimension (meaning any tangible piece of matter that we can see and work with in a laboratory).

## Section S4

***Calculating relative phase coordination dynamics***: For pilot test, to identify a relevant in-phase bi-manual synchrony variable, the collected data were analysed to compare the different characteristics between one joint performance and several different joint performances. Since the wrist point was more often used compared to other possible bi-manual pendulum areas such as the elbow or shoulder, whether the collected value is representative of the overall characteristics of a system must be assessed. Moreover, repeating the assessment for only one position under several different conditions and several trials is likely to be associated with learning (or fatigue) effects. Based on the obtained results, a method of data collection (excluding trial effects) was established in which a relevant dependent variable was used to measure the internal source of stability. Now, a question arises as to whether the data, which were taken from only one joint in several trials, well represents the characteristic of the system (see Table S1).

| Group | Participants (N) | Body joint | Trials | Task/rest (min) |
|---|---|---|---|---|
| G1 | 8 | Wrist | 6 | 1 m / 5 m |
| | | Wrist | 2 | 1 m / 5 m |
| G2 | 8 | Elbow | 2 | 1 m / 5 m |
| | | Shoulder | 2 | 1 m / 5 m |

**Table S1.** *Data collection for the pilot test 1:* Group one = 8 participants, 6 trials, at wrist (total dataset = 48). Group 2 = 8 participants, 3 joints, and 2 trials at each joint with random sequences (total dataset = 48). Duration of each trial is 1 minute, with 5-minute rest intervals between trials.

The sessions were divided into two, with different conditions (one joint = group 1, and different joint = group 2) but only one normal-temperature embedding cycle (5:00 pm - highest peak circadian rhythm of the core temperature with skin capacitance). Each trial block lasted 1 min with a resting period of 5 min. During the one joint session (wrist), participants received instructions regarding the preferred pendulum movements to establish in the in-phase 1:1 frequency locking at a 1.21 s metronome beat (this period was chosen because it corresponded to the natural period of the pendulum system [37]) without considering the amplitude or frequency. In the different joint session (wrist, elbow, and shoulder), the participants received instructions regarding the preferred pendulum, similar to the single-joint session, but with the additional instruction of keeping different joints voluntarily fixed. A small amount of experience was provided to overcome the challenges that arise when complying with the session requirements. During the actual trial, no feedbacks were given, and the participants were not required to report except when a problem arises. If the participants accidentally moved a joint that was supposed to be fixed voluntarily, the data from that trial were not analysed, and the trial was repeated later. The three different oscillation joints were used in a random order.

***Finding a Relevant In-phase Bi-manual Synchrony Variable (for the pilot test)***: Data were collected from 16 participants (at the University of Connecticut) (M=10, F=6, age 22 $\pm$ 3) under the normal condition (5:00 pm) for comparing a typical anatomical position (wrist: M=5, F=3) with several different joint positions (wrist, elbow, and shoulder: M=5, F=3). Participants were divided into different experimental groups and were encouraged to engage in bimanual coordination in-phase 1:1 frequency locking at a 1.21 s metronome beat for the following two reasons: (a) although the wrist point was the area that was the most commonly considered compared to other possible bi-manual pendulum areas, such as the elbow and shoulder, the value

must be able to represent the overall characteristic of the system in an effective manner. Moreover, (b) it was not clear as to whether repeating only one position under several different conditions through several trials had a link with learning (or fatigue) effects.

First, the investigation was divided into phases for different conditions according to the trial. Thereafter, the data were analysed up to two values under the same normal condition (5:00 pm). This was done based on which one would be better as the experimental dependent variable to illustrate the different trial effects between the two conditions of the one joint (wrist) or the position of different joints (wrist, elbow, and shoulder) (see Figure 4).

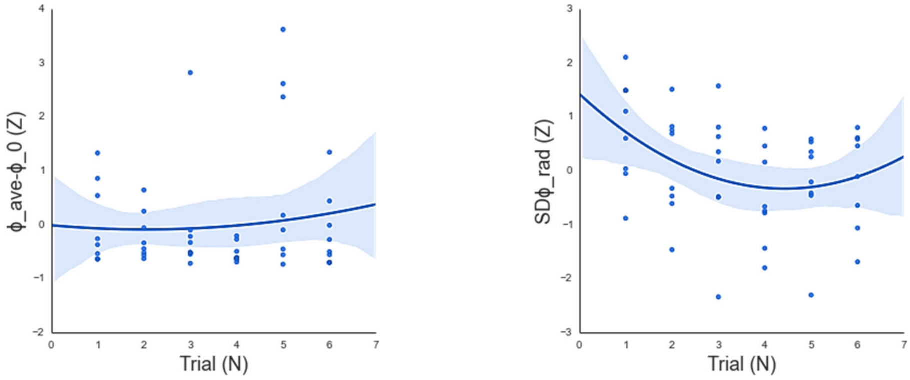


**Figure S4.1.** Repeated measure of the wrist according to the trial. Left = from the intended phase, $\phi_{ave} - \phi_0(rad)$, and right = standard deviation of the relative phase, $SD\phi(rad)$. Z is the standard score of the observed raw score $\chi$ (formula: Z = $\frac{\chi-\mu}{\sigma}$).

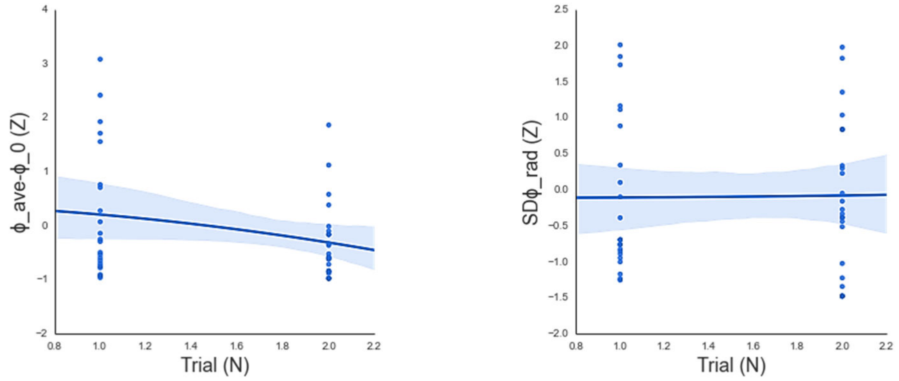


**Figure S4.2.** Repeated measure of the different joints (wrist, elbow, and shoulder) according to the trial. Left = from the intended phase, $\phi_{ave} - \phi_0(rad)$, and standard deviation of relative phase, $SD\phi(rad)$ = right. Z is the standard score of the observed raw score $\chi$ (formula: Z = $\frac{\chi-\mu}{\sigma}$).

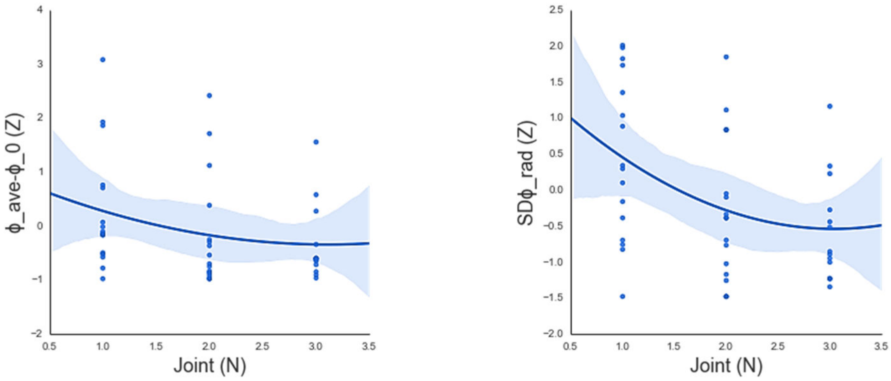


**Figure S4.3.** Deviation of the phase for the different joint orientation (1 = wrist, 2 = elbow, 3 = shoulder). Left denotes the topological effect [left = mean relative phase from the intended phase, $\phi_{ave} - \phi_0(rad)$, right = standard deviation of relative phase, $SD\phi(rad)$]. Z is the standard score of the observed raw score $\chi$ (formula: $Z = \frac{\chi-\mu}{\sigma}$). Note: In addition, we initially calculated the correlation considering the interaction between both values and found that both conditions have a significant relationship [$\phi_{ave} - \phi_0(rad)$] and [$SD\phi(rad)$]. This indicates that although the correlation functions were different according to the time series between [$\phi_{ave} - \phi_0(rad)$], [$\phi_{ave} - \phi_0(rad)$], [$SD\phi(rad)$], the higher [$\phi_{ave} - \phi_0(rad)$] correlated with higher [$SD\phi(rad)$] and lower [$\phi_{ave} - \phi_0(rad)$] also correlated with lower [$SD\phi(rad)$]: [Wrist Pearson Correlation $R = .46$ ($p = 0.0011$)], [Topology Pearson Correlation $R = .5$ ($p = 0.00041$)]. Such characteristics correspond to our predicted illustration of the relative phase based on the coordination dynamic calculations: $\phi_{ave} - \phi_0$ = fixed point shift, $SD\phi$ = variability as a function of frequency competition.

The results showed that motor performance varied with different anatomical parts given the shift of the mean relative phase from an intended phase [$\phi_{ave} - \phi_0(rad)$] and the in-phase variability [$SD\phi(rad)$] of the three behavioural variables. The main observation was that repetition with one joint may be significantly associated with decreased variability, akin to trial effects [Pearson Correlation $R = -.284$ ($p < 0.025$)], while the other condition (different topology) does not have a significant effect according to the trial [Pearson Correlation $R = .110$ ($p < 0.236$)]. However, there is hierarchical significance, as the mean relative phase from an intended phase [$\phi_{ave} - \phi_0(rad)$] and the variability [$SD\phi(rad)$] were significantly wider for distal anatomy: $F(2, 47) = 4656.999$, ($p < 0.001$).

This investigation specifically considers these differences to determine the fundamental characteristic of different anatomical joints (see Figure 5). Figure 5 illustrates the average stabilities of each joint for the wrist, elbow, and shoulder performances. Each topological asymmetry, coupling, and noise oscillation are reflecting, showing that they were significantly wider for the distal (wrist) than the proximal (shoulder) with different anatomical parameters. This characteristic of symmetrical bi-manual relationships may indicate greater heterogeneity of the scaling exponents in certain topological point.

Wrist Elbow Shoulder

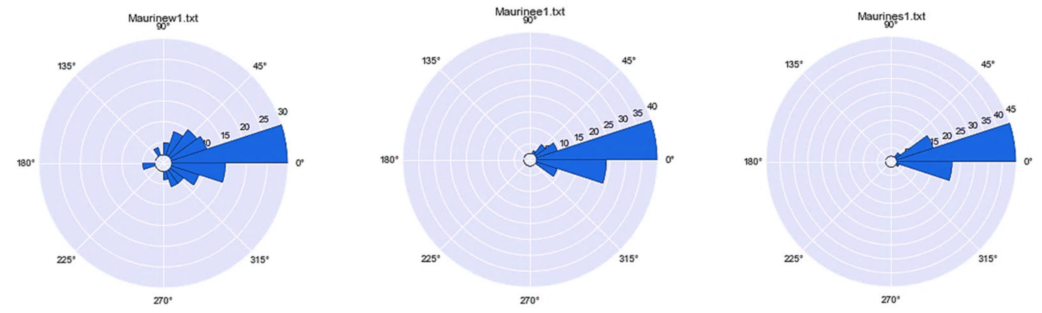

**Figure S5.1.** Circular representation of the different joint coupled oscillations: Left = wrist, middle = elbow, right = shoulder (shaded sector arear means pendulum angle degree and variance). Note: When it comes to these circular functions, we used 2π as a default (0) and calculated x using (180 degree*x/pi). In the above sample case, the degree represents the closeness to in phase (0 degree, or 360 degree) or anti phase (±180 degree) and variance (distribution) of each topological relative phase.

Wrist Elbow Shoulder

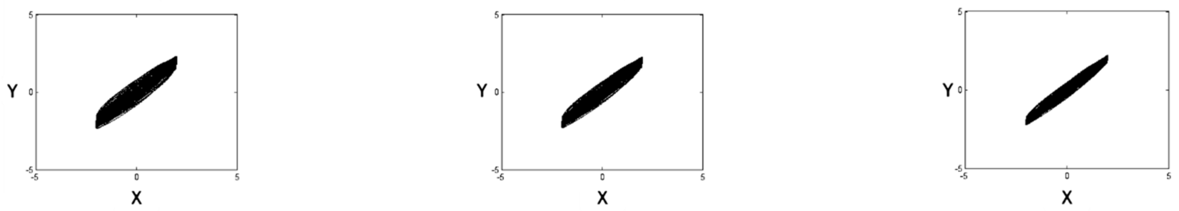

**Figure S5.2.** Coupling strength of the different joint coupled oscillations. This denotes all participants' range of oscillations. We used T score ($T = 10 * \frac{\chi-\mu}{\sigma} + 50$) as a different noise value (left: wrist = 61.476, middle: elbow = 51.419, right: shoulder = 37.105), and same coupling strength = 1 (center line at each scope). X_Y plots illustrate the relationship between each oscillator's horizontal(x)-axis and vertical(x)-axis patterns [left: wrist, middle: elbow, right: shoulder] visualizing two-dimensional noise (x_axis = variance of group SD, y_axis = variance of group M).

Inspired by these analyses, collecting different values for three joints while using only wrist data for biological characterization was determined as an ideal approach for the following reasons. (a) Although a significant learning effect was present when the participants undertook a task repeatedly with only the wrist point, significant typicality was noticed as compared to the other two (elbow and shoulder) datasets. As such, to overcome the learning (or fatigue) effects, the data were collected for the three joints randomly, however, only the wrist data were used. (b) Although combined data including all the three different motor positions could have been used, representing the data as the characteristic of a biological system could have eliminated the importance of representativeness, as such combinations likely give rise to too many variables, which can sometimes be difficult to manage. Moreover, (c) there was an expectation that collecting different motor scales but using widely represented data (wrist position) may meet both the requirements of typicality as a well-defined system characteristic and the elimination of the learning effect stemming from numerous trials. Thus, the aforementioned approach was chosen to represent a typical internal source (dependent variable).

## Section S5

***Normal and abnormal day–night circadian temperature perturbations***: There is a state of unit $x$ at time t $[x(t)]$ and this denotes the ensemble average of the system. Collective behavior emerges from a homogeneous state when a parameter makes a transition from $x = 0$ to $x \neq 0$. To reflect this transition in terms of an external parameter, the temperature is such that all terms of the parameter which can be considered as perturbations are considered in this case.

$$\epsilon\ (\chi, t) = \mathrm{T}\ (\chi, t) - \ \mathrm{T}_0(t) \tag{17}$$

Here, $\epsilon$ is considered as the control parameter while is T fixed. However, it is useful to capture the effective control parameter $\mathrm{T}_0$ to measure the distance of $\epsilon$ with respect to T. That is, the $\mathrm{T}_0 < \epsilon$ state is unstable (ice-vested and heat-vested), whereas the $\mathrm{T}_0 > \epsilon$ state is stable (normal).

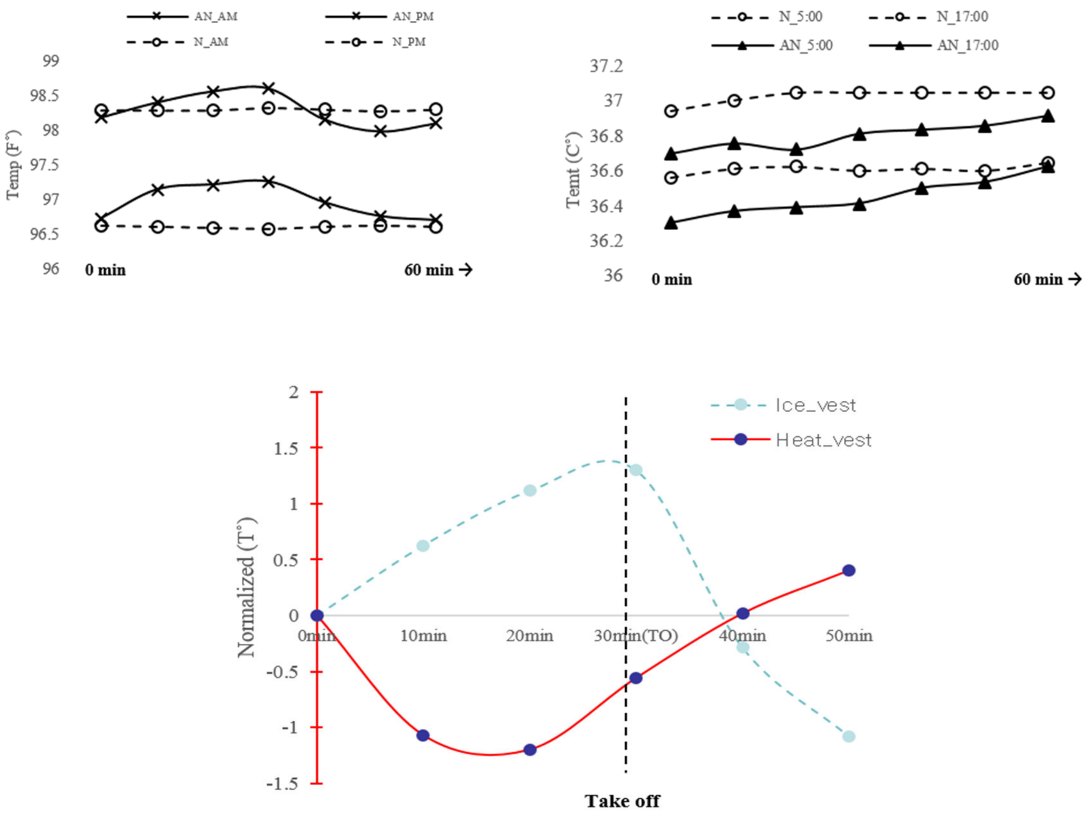


**Figure S6.1.** Plots show illustration of the temperature according to the experimental design: normal (dotted line) and abnormal (dashed line) conditions. The horizontal axis denotes the temperature check time (TO = take off the ice/heat vest under the perturbation condition). The vertical axis is the level of the temperature change as calculated in Fahrenheit (F°) and Celsius (C°) degrees. Upper left = separate temperatures between the normal (N: AM and PM) and heat-based abnormal (AN: AM and PM) conditions, upper right = separate temperatures between the normal (N: AM and PM) and ice-based abnormal (AN: AM and PM) conditions. Note: in the abnormal session, the data were collected after 30 minutes (from 30 minutes to 60 minutes) of taking the ice (or heat) vest off, while in the normal session, the data were collected after 30 minutes without taking the ice (or heat) vest off in order to ensure that the conditions were identical. Bottom

= normalized temperature adaptation tendency according to the artificially managed perturbation (the dotted line denotes the ice-vest perturbation; the bolded line denotes the heat-vest perturbation).

This temperature perturbation would be interpreted as a thermodynamic variable; that is, it is not restricted by itself to the usual set of thermodynamic variables, such as the mean internal energy and entropy.

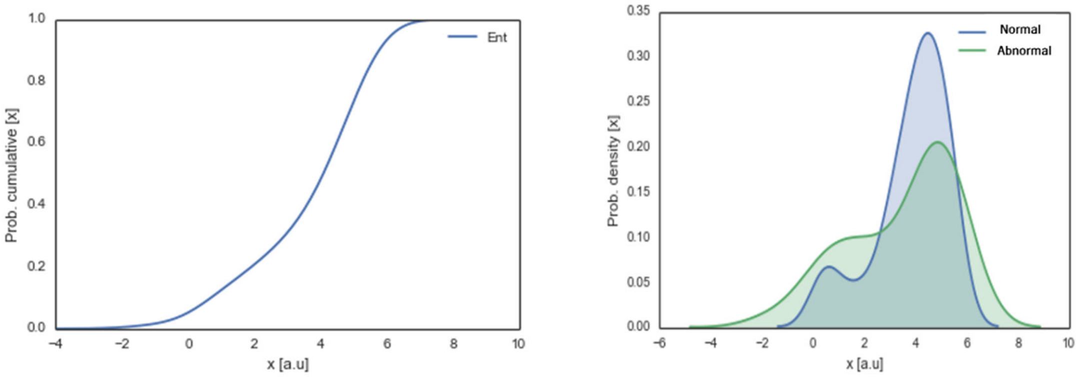


**Figure S6.2.** Plots show estimated entropy production depending on the perturbation. The left side denotes the nominal distribution of all cases of entropy (x) production with a cumulative function [proportion (vertical axis) of the entropy value (horizontal axis)] with the arbitrary unit (a.u.). The right side represents the density of the entropy (data double plotted kernel density function) according to the temperature perturbation (false = normal, true = abnormal).

As shown in Figure above, the emergence of the collective behavior of the increasing distance between $\mathrm{T}\,(\chi, t) - \mathrm{T}_0(t)$ via a perturbation was related to an increase (or decrease) in the entropy. Comparing the states of $\mathrm{T}_0 < \epsilon$ and $\mathrm{T}_0 > \epsilon$, as shown in Figure 26 on the right, as the distance ordered state becomes smaller, the peak in the entropy production rate becomes higher. It is also important to note that the biological non-equilibrium bias towards a different temperature component hints at a possible deep connection between physical stability and entropy production. Given the above entropy production embedded in the order-disorder biophysical dynamic, the differences between the two circadian rhythm (termed a nearly 24-h instance of oscillatory variation) points (AM and PM) and the difference between the two temperature conditions to the psychomotor vigilances (i.e., $\dot{\phi}$ biological motor stability) were compared to determine whether differences in biological disorder result in differences in environmental perturbations.

According to the statistical testing of the data with direct reference to the research questions or hypotheses, the value of the equation "entropy = H (x)" was estimated considering the following null ($H_0$: $\theta_A = \theta_B$) and alternative ($H_a$: $\theta_A \neq \theta_B$) hypotheses. The hypothesis was proved that different experimental conditions of the external source have a significant effect on the internal source (bi-manual motor variable) of $\dot{\phi}$. More specifically, different external components of the circadian processes or temperature have a significant effect on the internal stability; and the internal perturbation, from an external source, will have a significant effect on the degree of biological entropy. Specifically, the statistic $F$ is calculated by dividing the difference between the group ($MS_{between}$) value by the difference between the value for the subjects within the group ($MS$). Observations are interested in the main effect of the circadian rhythm ($\alpha$), the temperature perturbation ($\beta$), and the interaction between the circadian rhythm and the temperature perturbation ($\alpha \times \beta$) as it affects the dependent variable of entropy production. Thus, the $F$ distribution was compared associated with each feature of interest to the error variance to determine if each effect is meaningful.

## Section S6

Each type of entropy value in normal day-night temperature effects.

| | | Circadian 5:00 | Circadian 12:00 | Circadian 17:00 | Circadian 00:00 |
|---|---|---|---|---|---|
| P1 | Trial_W1 | 4.073 | 2.965 | 4.017 | 5.783 |
| | Trial_W2 | 3.785 | 2.800 | 4.030 | 5.856 |
| | *I* | 3.929 | 2.882 | 4.023 | 5.819 |
| P2 | Trial_W1 | 5.813 | 5.492 | 3.623 | 5.721 |
| | Trial_W2 | 5.846 | 5.210 | 4.459 | 5.033 |
| | *I* | 5.830 | 5.351 | 4.041 | 5.377 |
| P3 | Trial_W1 | 4.216 | 3.849 | 3.741 | 5.714 |
| | Trial_W2 | 4.171 | 3.790 | 3.642 | 3.741 |
| | *I* | 4.194 | 3.820 | 3.691 | 4.728 |
| P4 | Trial_W1 | 5.877 | 5.877 | 4.806 | 3.328 |
| | Trial_W2 | 5.883 | 5.900 | 4.234 | 4.469 |
| | *I* | 5.880 | 5.888 | 4.520 | 3.898 |
| P5 | Trial_W1 | 5.688 | 5.620 | 3.218 | 5.809 |
| | Trial_W2 | 5.869 | 4.888 | 5.644 | 2.817 |
| | *I* | 5.779 | 5.254 | 4.431 | 4.313 |
| P6 | Trial_W1 | 5.887 | 5.866 | 5.726 | 5.787 |
| | Trial_W2 | 3.937 | 5.841 | 5.815 | 5.808 |
| | *I* | 4.912 | 5.853 | 5.771 | 5.798 |
| P7 | Trial_W1 | 5.683 | 5.773 | 3.174 | 5.865 |
| | Trial_W2 | 5.831 | 5.477 | 4.943 | 5.792 |
| | *I* | 5.757 | 5.625 | 4.059 | 5.829 |
| P8 | Trial_W1 | 5.653 | 4.943 | 5.835 | 5.840 |
| | Trial_W2 | 5.724 | 5.868 | 5.795 | 5.838 |
| | *I* | 5.688 | 5.405 | 5.815 | 5.839 |

*Notes*. P is the participant with each number of 1 ~ 8, Trial_W1 and 2 denotes the actually used wrist data set from three different joint data sets, H (x) = entropy production. Note: the value of *I* is derived from the execution of each trial ($w_1$, $w_2$), these two trials' value were divided by 2.

Each type of entropy value in normal and abnormal (heat based) day-night temperature effects.

| | | N_5:00 Normalized (Z) | N_17:00 Normalized (Z) | Ab_5:00 Normalized (Z) | Ab_17:00 Normalized (Z) |
|---|---|---|---|---|---|
| P1 | Trial_W1 | -0.549 | -0.595 | 0.861 | -1.056 |
| | Trial_W2 | -0.785 | -0.585 | 0.909 | -0.932 |
| | *I* | -0.667 | -0.590 | 0.885 | -0.994 |
| P2 | Trial_W1 | 0.873 | -0.918 | 0.909 | -0.777 |
| | Trial_W2 | 0.900 | -0.234 | 0.897 | -1.034 |
| | *I* | 0.887 | -0.576 | 0.903 | -0.906 |
| P3 | Trial_W1 | -0.433 | -0.821 | 0.689 | -0.045 |
| | Trial_W2 | -0.469 | -0.902 | -0.712 | -1.144 |
| | *I* | -0.451 | -0.862 | -0.012 | -0.595 |
| P4 | Trial_W1 | 0.926 | 0.050 | 0.832 | -2.624 |
| | Trial_W2 | 0.930 | -0.418 | 0.916 | -2.044 |
| | *I* | 0.928 | -0.184 | 0.874 | -2.334 |
| P5 | Trial_W1 | 0.771 | -1.249 | -0.473 | -1.859 |
| | Trial_W2 | 0.919 | 0.735 | -1.086 | 0.829 |
| | *I* | 0.845 | -0.257 | -0.780 | -0.515 |
| P6 | Trial_W1 | 0.934 | 0.802 | 0.934 | 0.678 |
| | Trial_W2 | -0.661 | 0.875 | 0.896 | -2.856 |
| | *I* | 0.137 | 0.839 | 0.915 | -1.089 |
| P7 | Trial_W1 | 0.767 | -1.285 | 0.920 | -1.285 |
| | Trial_W2 | 0.888 | 0.162 | 0.863 | 0.881 |
| | *I* | 0.828 | -0.561 | 0.892 | -0.202 |
| P8 | Trial_W1 | 0.742 | 0.891 | 0.925 | 0.278 |
| | Trial_W2 | 0.801 | 0.858 | 0.742 | 0.045 |
| | *I* | 0.771 | 0.875 | 0.834 | 0.162 |

*Notes.* P is the participant with each number of 1 ~ 8, Trial_W1 and 2 denotes the actually used wrist data set from three different joint data sets. N denotes normal circadian condition, and Ab denotes heat_vested abnormal circadian condition. Note: In order to more dramatic visualization, we used standard score (Z calculation). The value of *I* is derived from the execution of each trial ($w_1$, $w_2$), these two trials' value were divided by 2.

Each type of entropy value in normal and abnormal (ice based) day-night temperature effects.

| | | N_5:00 Normalized (Z) | N_17:00 Normalized (Z) | Ab_5:00 Normalized (Z) | Ab_17:00 Normalized (Z) |
|---|---|---|---|---|---|
| P1 | Trial_W1 | 0.453 | -1.906 | 0.601 | -1.921 |

| | | | | | |
|---|---|---|---|---|---|
| | Trial_W2 | 0.321 | -1.906 | -1.235 | -1.918 |
| | *I* | 0.387 | -1.906 | -0.317 | -1.920 |
| P2 | Trial_W1 | 0.455 | -0.002 | 0.829 | -1.819 |
| | Trial_W2 | 1.015 | 1.019 | -0.835 | -1.888 |
| | *I* | 0.735 | 0.508 | -0.003 | -1.853 |
| P3 | Trial_W1 | 0.679 | -0.386 | 1.084 | -2.066 |
| | Trial_W2 | 0.367 | -0.450 | 1.016 | -1.941 |
| | *I* | 0.523 | -0.418 | 1.050 | -2.004 |
| P4 | Trial_W1 | 1.000 | 1.037 | 0.929 | 0.613 |
| | Trial_W2 | 0.958 | 0.694 | 0.929 | 0.018 |
| | *I* | 0.979 | 0.865 | 0.929 | 0.315 |
| P5 | Trial_W1 | 0.329 | 0.152 | 0.298 | -0.075 |
| | Trial_W2 | 0.299 | -0.160 | 1.160 | -1.000 |
| | *I* | 0.314 | -0.004 | 0.729 | -0.537 |
| P6 | Trial_W1 | -0.277 | 0.589 | 1.190 | 0.512 |
| | Trial_W2 | 0.794 | 0.294 | 0.925 | -0.565 |
| | *I* | 0.259 | 0.442 | 1.057 | -0.027 |
| P7 | Trial_W1 | -0.173 | -1.770 | 0.871 | -1.090 |
| | Trial_W2 | -0.898 | -1.241 | 1.028 | -0.856 |
| | *I* | -0.535 | -1.505 | 0.950 | -0.973 |
| P8 | Trial_W1 | 0.077 | 0.753 | 0.054 | 0.363 |
| | Trial_W2 | 1.071 | 0.525 | 0.882 | 0.193 |
| | *I* | 0.574 | 0.639 | 0.468 | 0.278 |

Notes. P is the participant with each number of 1 ~ 8, Trial_W1 and 2 denotes the actually used wrist data set from three different joint data sets. N denotes normal circadian condition, and Ab denotes ice_vested abnormal circadian condition. Note: In order to more dramatic visualization, we used standard score (Z calculation). The value of *I* is derived from the execution of each trial ($w_1$, $w_2$), these two trials' value were divided by 2.
